# Delirium in Older Patients With COVID-19 Presenting to the Emergency Department

**DOI:** 10.1001/jamanetworkopen.2020.29540

**Published:** 2020-11-19

**Authors:** Maura Kennedy, Benjamin K. I. Helfand, Ray Yun Gou, Sarah L. Gartaganis, Margaret Webb, J. Michelle Moccia, Stacey N. Bruursema, Belinda Dokic, Brigid McCulloch, Hope Ring, Justin D. Margolin, Ellen Zhang, Robert Anderson, Rhonda L. Babine, Tammy Hshieh, Ambrose H. Wong, R. Andrew Taylor, Kathleen Davenport, Brittni Teresi, Tamara G. Fong, Sharon K. Inouye

**Affiliations:** 1 Department of Emergency Medicine, Massachusetts General Hospital, Boston; 2 Department of Emergency Medicine, Harvard Medical School, Boston, Massachusetts; 3 Department of Emergency Medicine, University of Massachusetts Medical School, Worcester; 4 Department of Psychiatry and Human Behavior and Neurology, Warren Alpert Medical School, Brown University, Providence, Rhode Island; 5 Department of Neurology, Warren Alpert Medical School, Brown University, Providence, Rhode Island; 6 Aging Brain Center, Marcus Institute for Aging Research, Hebrew SeniorLife, Harvard Medical School, Boston, Massachusetts; 7 Emergency Medicine, St Mary Mercy Livonia Hospital, Livonia, Michigan; 8 Department of Neurology, Beth Israel Deaconess Medical Center, Harvard Medical School, Boston, Massachusetts; 9 Department of Emergency Medicine, Maine Medical Center, Portland; 10 Department of Clinical Nursing Resources, Maine Medical Center, Portland; 11 Department of Medicine, Brigham and Women’s Hospital, Harvard Medical School, Boston, Massachusetts; 12 Department of Emergency Medicine, Yale School of Medicine, New Haven, Connecticut; 13 Department of Emergency Medicine, University of North Carolina at Chapel Hill School of Medicine, Chapel Hill; 14 Department of Medicine, Beth Israel Deaconess Medical Center, Harvard Medical School, Boston, Massachusetts

## Abstract

**Importance:**

Delirium is common among older emergency department (ED) patients, is associated with high morbidity and mortality, and frequently goes unrecognized. Anecdotal evidence has described atypical presentations of coronavirus disease 2019 (COVID-19) in older adults; however, the frequency of and outcomes associated with delirium in older ED patients with COVID-19 infection have not been well described.

**Objective:**

To determine how frequently older adults with COVID-19 present to the ED with delirium and their associated hospital outcomes.

**Design, Setting, and Participants:**

This multicenter cohort study was conducted at 7 sites in the US. Participants included consecutive older adults with COVID-19 presenting to the ED on or after March 13, 2020.

**Exposure:**

COVID-19 was diagnosed by positive nasal swab for severe acute respiratory syndrome coronavirus 2 (99% of cases) or classic radiological findings (1% of cases).

**Main Outcomes and Measures:**

The primary outcome was delirium as identified from the medical record according to a validated record review approach.

**Results:**

A total of 817 older patients with COVID-19 were included, of whom 386 (47%) were male, 493 (62%) were White, 215 (27%) were Black, and 54 (7%) were Hispanic or Latinx. The mean (SD) age of patients was 77.7 (8.2) years. Of included patients, 226 (28%) had delirium at presentation, and delirium was the sixth most common of all presenting symptoms and signs. Among the patients with delirium, 37 (16%) had delirium as a primary symptom and 84 (37%) had no typical COVID-19 symptoms or signs, such as fever or shortness of breath. Factors associated with delirium were age older than 75 years (adjusted relative risk [aRR], 1.51; 95% CI, 1.17-1.95), living in a nursing home or assisted living (aRR, 1.23; 95% CI, 0.98-1.55), prior use of psychoactive medication (aRR, 1.42; 95% CI, 1.11-1.81), vision impairment (aRR, 1.98; 95% CI, 1.54-2.54), hearing impairment (aRR, 1.10; 95% CI 0.78-1.55), stroke (aRR, 1.47; 95% CI, 1.15-1.88), and Parkinson disease (aRR, 1.88; 95% CI, 1.30-2.58). Delirium was associated with intensive care unit stay (aRR, 1.67; 95% CI, 1.30-2.15) and death (aRR, 1.24; 95% CI, 1.00-1.55).

**Conclusions and Relevance:**

In this cohort study of 817 older adults with COVID-19 presenting to US emergency departments, delirium was common and often was seen without other typical symptoms or signs. In addition, delirium was associated with poor hospital outcomes and death. These findings suggest the clinical importance of including delirium on checklists of presenting signs and symptoms of COVID-19 that guide screening, testing, and evaluation.

## Key Points

**Question:**

How frequently do older adults (aged ≥65 years) with coronavirus disease 2019 (COVID-19) present to the emergency department (ED) with delirium?

**Findings:**

In this cohort study of 817 older ED patients with COVID-19, 28% had delirium at presentation, and delirium was the sixth most common of all presenting symptoms and signs. Among delirious patients, 16% presented with delirium as a primary symptom and 37% had no typical COVID-19 symptoms or signs, such as cough or fever.

**Meaning:**

These findings suggest that older adults with COVID-19 commonly present to the ED with delirium and that delirium should be considered an important presenting symptom of COVID-19.

## Teaser

This cohort study examines how frequently older adults with coronavirus disease 2019 (COVID-19) present to the emergency department with delirium and their associated hospital outcomes.

## Introduction

Severe acute respiratory syndrome coronavirus 2 (SARS-CoV-2), the novel coronavirus that causes coronavirus disease 2019 (COVID-19), was declared a pandemic on March 11, 2020, by the World Health Organization^1^ and a national emergency in the US on March 13, 2020.^2^ As of September 14, 2020, there were 29 114 477 confirmed cases of COVID-19 with 925 596 fatalities worldwide.^3^ Although SARS-CoV-2 poses a risk at all ages, adults aged 65 years and older are at greatest risk of severe disease, hospitalization, intensive care use, and death.^4^ Persons older than 65 years comprise 16% of the US population^5^ yet have accounted for more than 80% of deaths in the US.^6^

Beyond COVID-19, delirium is known to be a common presenting symptom for older adults with severe disease in the emergency department (ED) but goes undetected in two-thirds of cases.^7,8^ Delirium is an acute state of confusion, characterized by altered level of consciousness, disorientation, inattention, and other cognitive disturbances, that commonly affects older persons and is associated with adverse outcomes, including prolonged hospitalization and death.^9,10^ In non–COVID-19 illness, delirium may be the earliest, or only, sign of an infection.^11^ In older adults infected by SARS-CoV-2, anecdotal evidence and case series have described atypical presentations—namely, patients presenting without the typical signs or symptoms of an illness or with nonspecific signs and symptoms.^12^ For example, older persons can present with delirium in the absence of typical signs and symptoms of COVID-19, such as fever or cough.^13,14,15,16,17^ Early COVID-19 studies have estimated rates of delirium at 25% to 33% in hospitalized patients^18,19^ and 65% in intensive care unit (ICU) patients.^20^ In one study,^21^ among 113 patients with COVID-19 who died, 22% presented with altered level of consciousness, compared with 1% of those who recovered. The frequency of and outcomes associated with delirium among older adults with COVID-19 infection in the ED setting, however, have not been well described, to our knowledge. This information is critical to ensure early recognition of COVID-19 by frontline clinicians. The primary objective of this descriptive study was to determine the frequency of delirium as a presenting symptom of COVID-19 infection among older adults in the ED.

## Methods

### Overall Study Description

We conducted a multicenter cohort study evaluating consecutive older adults (aged ≥65 years) presenting to the ED and diagnosed with COVID-19 infection. Seven ED study sites were selected from across the US and included EDs in the Northeast (Boston, Massachusetts; Portland, Maine; and New Haven, Connecticut), Midwest (Livonia, Michigan), and South (Chapel Hill and Hillsborough, North Carolina). Each site reviewed and extracted deidentified data for consecutive older patients presenting to the ED on or after March 13, 2020 (the date COVID-19 was declared a national emergency in the US), and diagnosed with COVID-19, and continued reviewing consecutive medical records until either all medical records for COVID-19–positive patients were reviewed or at least 30 patients with delirium were included from that site. The period for which patients were enrolled varied by site (range, 3-11 weeks) because of variations in COVID-19 prevalence and ED patient volume.

Each study site secured relevant study approvals with waiver of informed consent, because the data were deidentified, or a determination of exemption from review by its institutional review board. The study was deemed as exempt by the institutional review board at Hebrew SeniorLife, the study coordinating center. This study follows Strengthening the Reporting of Observational Studies in Epidemiology (STROBE) reporting guideline.

### Study Population

We enrolled consecutive patients aged 65 years or older presenting to the ED and diagnosed with active COVID-19 infection, which was defined by either positive nasal swab polymerase chain reaction (PCR) test for SARS-CoV-2 (99% of cases) or chest radiograph or computed tomography demonstrating classic COVID-19 appearance and distribution of ground-glass opacities (1% of cases). We allowed the inclusion of high-probability radiographic diagnosis of COVID-19 because of national shortages in PCR tests; all but 4 of these cases were confirmed by positive SARS-CoV-2 test results.

### Approach

Data were extracted using a standardized data abstraction form. For consistency in data abstraction across locations, site investigators attended training sessions led by the principal investigator (S.K.I.), and centralized training documents were distributed. Data abstraction was performed by site investigators, who were experienced clinicians, or trained research assistants under close supervision.

The primary outcome was the presence of delirium at presentation to the ED. Delirium was identified through a reliable and widely used medical record review approach, with a sensitivity of 74%, specificity of 83%, likelihood ratio of 4.4, overall agreement of 82% (κ = 0.41) for delirium diagnoses, as validated against the Confusion Assessment Method,^22^ and high interrater reliability (κ = 1.0).^23^ This method has been used in many prior studies.^19,23,24,25,26,27^ The approach involves a detailed review of all physician, nursing, and other notes for presence of keywords related to delirium and evidence of acute change from baseline. Diagnosis codes for delirium are noted, but the rating is not based on these alone. All potential delirium symptoms and signs were recorded, and any uncertain cases were adjudicated by at least 2 clinical experts. Some sites routinely used delirium screening tools, which aided in delirium detection. In addition to the presence of delirium and how it was identified, we recorded delirium duration before presentation.

A checklist of the presence of any of 25 symptoms and signs associated with COVID-19 infection, derived from the Centers for Disease Control and Prevention (CDC), World Health Organization case report checklists, and epidemiological studies, was used to record specific presenting symptoms and signs for each patient. Other data collected included the specific method of COVID-19 diagnosis, patient demographic characteristics (age, sex, race/ethnicity, English-language fluency, education, and living situation), preadmission delirium risk factors (prior use of psychoactive medications, alcohol, smoking, and any of 14 major comorbid illnesses documented in the medical record, including cognitive impairment or dementia, stroke, Parkinson disease, chronic kidney disease, and sensory impairment), and clinical outcomes, including hospitalization rates, ICU stay, use of mechanical ventilation, hospital length of stay, hospital disposition, and mortality. Data were entered for electronic data capture via SurveyGizmo Premium software (Alchemer). No identifying data were collected, and age older than 90 years was recorded as greater than or equal to 90 years. All uploaded data were blinded to specific site location.

### Statistical Analysis

For baseline characteristics and symptoms at presentation, standard descriptive statistics were reported. To analyze factors associated with risk of delirium, we used generalized linear models assuming a Poisson distribution with a log-link function to estimate the relative risk (RR) of delirium with robust SEs. We used the generalized linear models approach because it provides stable estimates even with small sample sizes and direct estimation of RRs.^28^ After examining distributions, each risk factor was first examined in an unadjusted model, and then all significant risk factors were examined together in a multivariable model. Because of a high rate of missing data, education was removed from the multivariable model. Variables that demonstrated high multicollinearity were removed, yielding the final multivariable risk factor model. To analyze outcomes associated with delirium, the RR of outcome events in the presence (vs absence) of delirium was estimated in both unadjusted and multivariable generalized linear models, adjusted for age older than 75 years, the presence of 4 or more comorbid conditions, nursing home or assisted living residency, and use of psychoactive medications. Missing data, not reported in the medical record, were present for only 4 variables: education (627 patients [77%]), race/ethnicity (30 patients [4%]), English fluency (16 patients [2%]), and hospital length of stay (1 patient [<1%]). The small numbers of patients with missing data were excluded from the relevant risk factor analyses (eg, for race and English fluency), and 1 person with missing data was excluded from the length of stay outcome analysis. All statistical analyses were conducted with Stata statistical software version 15 (StataCorp). Significance testing for all analyses was 2-sided with a type I error of .05.

## Results

A total of 817 patients were examined, of whom 386 (47%) were male, 493 (62%) were White, 215 (27%) were Black, and 54 (7%) were Hispanic or Latinx (Table 1). The mean (SD) age of study participants was 77.7 (8.2) years, and 213 (26%) resided in a skilled nursing facility.

**Table 1.** Characteristics of Older Emergency Department Patients With Coronavirus Disease 2019

Characteristic	Patients, No. (%) (N = 817)
Age, mean (SD), y	77.7 (8.2)
Male	386 (47)
Race/ethnicity^a^	
White	493 (62)
Black	215 (27)
Hispanic or Latinx	54 (7)
Asian	12 (2)
Multiracial	19 (2)
Not reported	24 (3)
Education less than high school	34 (18)
English, nonfluent	111 (14)
Living in nursing home	213 (26)
Living in assisted living	84 (10)

^a^ Multiple categories were allowed, so the total percentage exceeds 100%.

Table 2 shows the most common COVID-19 symptoms and signs reported on presentation to the ED, including fever (459 patients [56%]), shortness of breath (420 patients [51%]), cough (412 patients [50%]), hypoxia (324 patients [40%]), and weakness (241 patients [30%]). A total of 226 patients (28%) had delirium at presentation, and delirium was the sixth most common of all presenting symptoms and signs. Most cases of delirium were identified either from physician (178 patients [79%]) or nursing (133 patients [59%]) assessments. Of the cases of delirium identified through the review of the medical records, only 22% of diagnoses (for 50 patients) were documented using a delirium assessment tool; however, this is likely an underestimate because clinicians may have used a tool without specific medical record documentation. Of patients with delirium, 37 (16%) had delirium as a primary presenting complaint. Importantly, among patients with delirium, 84 (37%) had no fever or shortness of breath. Common symptoms of delirium recorded in the medical record (Table 2) included impaired consciousness (122 patients [54%]), disorientation (96 patients [43%]), hypoactive delirium symptoms (45 patients [20%]), and agitation or hyperactive delirium symptoms (35 patients [16%]). Of all patients who had delirium, 112 (50%) presented to the ED within 2 days of onset of delirium and the vast majority (213 patients [94%]) were hospitalized.

**Table 2.** Symptoms and Signs at Emergency Department Presentation in Older Adults With Coronavirus Disease 2019

Symptoms	Patients, No. (%) (N = 817)
Symptom at presentation	
Fever	459 (56)
Shortness of breath	420 (51)
Cough	412 (50)
Hypoxia (oxygen saturation <90%)	324 (40)
Weakness	241 (30)
Delirium	226 (28)
Fatigue	210 (26)
Diarrhea	130 (16)
Anorexia	122 (15)
Myalgias	99 (12)
Fall	91 (11)
Dizziness, fainting	70 (9)
Abdominal pain	66 (8)
Acute respiratory failure	54 (7)
Headache	51 (6)
Chest pain	50 (6)
Sore throat	48 (6)
Other symptoms^a^	106 (13)
Delirium at presentation (n = 226)	
Delirium as main or primary presenting symptom	37 (16)
Delirium present without fever or shortness of breath	84 (37)
Approach for delirium identification	
Delirium assessment tool	50 (22)
Physician assessment	178 (79)
Nursing assessment	133 (59)
Delirium symptoms present	
Impaired consciousness	122 (54)
Disorientation	96 (43)
Inattention	71 (31)
Disorganized thinking	59 (26)
Hypoactive delirium	45 (20)
Agitation or hyperactive delirium	35 (16)
Memory loss	18 (8)
Hallucinations	7 (3)
Duration of delirium symptoms before emergency department presentation	
<2 d	112 (50)
Between 2 d to 1 wk	60 (27)
>1 wk	8 (4)
Not reported	36 (16)

^a^ Other symptoms (each reported in <5% of cases) included injury, dehydration, gait disturbance, acute impairment in taste, smell, or vision, acute cerebrovascular event, microembolic phenomenon, and seizure.

Table 3 presents factors potentially associated with risk of delirium. Significant prebaseline risk factors included age older than 75 years (459 patients [56%]), prior psychoactive medications (464 patients [57%]), and living in a nursing home or assisted living (297 patients [36%]). Preexisting comorbidities associated with increased risk for delirium included vision impairment (59 patients [7%]), hearing impairment (54 patients [7%]), cognitive impairment or dementia (248 patients [30%]), history of stroke or cerebrovascular accident (107 patients [13%]), Parkinson disease (23 patients [3%]), and mental health conditions (227 patients [28%]). The presence of 4 or more of the 14 chronic comorbidities was associated with a significant 1.58-fold increased risk for delirium (RR, 1.58; 95% CI, 1.23-2.04). There was no significant difference in delirium rates by sex or race/ethnicity groups.

**Table 3.** Risk Factors and Correlates of Delirium in Older Emergency Department Patients With Coronavirus Disease 2019

Risk factor	Risk factor prevalence, patients, No. (%) (N = 817)	Patients with delirium, No./total (%)		RR (95% CI)
		Risk factor present	Risk factor absent	
Risk factors (before baseline)^a^				
Age >75 y	459 (56)	157/459 (34)	69/358 (19)	1.77 (1.39-2.27)
Male	386 (47)	103/386 (27)	123/431 (29)	0.94 (0.75-1.17)
Black	215 (27)	58/215 (27)	164/578 (28)	0.95 (0.74-1.23)
Hispanic or Latinx	54 (7)	11/54 (20)	211/739 (29)	0.71 (0.42-1.22)
Nonwhite race	300 (38)	79/300 (26)	143/493 (29)	0.91 (0.72-1.15)
English, nonfluent	111 (14)	29/111 (26)	193/690 (28)	0.93 (0.67-1.31)
Living in nursing home	213 (26)	72/213 (34)	154/604 (26)	1.33 (1.05-1.67)
Living in nursing home or assisted living	297 (36)	104/297 (35)	122/520 (24)	1.49 (1.20-1.86)
Heavy alcohol use	20 (2)	4/20 (20)	222/797 (28)	0.72 (0.30-1.74)
Active smoker	16 (2)	5/16 (31)	221/801 (28)	1.13 (0.54-2.36)
Prior psychoactive medication use	464 (57)	155/464 (33)	71/353 (20)	1.66 (1.30-2.12)
Chronic conditions (comorbidities)				
≥4 Chronic conditions	119 (15)	48/119 (40)	178/698 (26)	1.58 (1.23-2.04)
Vision impairment	59 (7)	36/59 (61)	190/758 (25)	2.43 (1.92-3.09)
Hearing impairment	54 (7)	23/54 (43)	203/763 (27)	1.60 (1.15-2.23)
Cognitive impairment or dementia	248 (30)	95/248 (38)	131/569 (23)	1.66 (1.34-2.07)
Stroke or cerebrovascular accident	107 (13)	46/107 (43)	180/710 (25)	1.70 (1.32-2.18)
Parkinson disease	23 (3)	14/23 (61)	212/794 (27)	2.28 (1.61-3.23)
Chronic pulmonary disease	221 (27)	53/221 (24)	173/596 (29)	0.83 (0.63-1.08)
Diabetes	312 (38)	90/312 (29)	136/505 (27)	1.07 (0.85-1.34)
Obesity (body mass index ≥35)^b^	67 (8)	21/67 (31)	205/750 (27)	1.15 (0.79-1.67)
Active cancer	37 (5)	10/37 (27)	216/780 (28)	0.98 (0.57-1.68)
Chronic kidney disease	179 (22)	51/179 (29)	175/638 (27)	1.04 (0.80-1.35)
Requiring hemodialysis	38 (5)	8/38 (21)	218/779 (28)	0.75 (0.40-1.41)
Chronic liver disease	23 (3)	8/23 (35)	218/794 (28)	1.27 (0.72-2.24)
Immunosuppressive conditions	69 (8)	14/69 (20)	212/748 (28)	0.72 (0.44-1.16)
Mental health conditions	227 (28)	76/227 (34)	150/590 (25)	1.32 (1.05-1.66)

Abbreviation: RR, relative risk.

^a^ Data are missing on race variables for 3% of cases and on English fluency for 2% of cases.

^b^ Body mass index is calculated as weight in kilograms divided by height in meters squared.

The final multivariable risk factor model consisted of age older than 75 (adjusted RR [aRR], 1.51; 95% CI, 1.17-1.95), nursing home or assisted living facility residence (aRR, 1.23; 95% CI, 0.98-1.55), prior use of psychoactive medications (aRR, 1.42; 95% CI, 1.11-1.81), vision impairment (aRR, 1.98; 95% CI, 1.54-2.54), hearing impairment (aRR, 1.10; 95% CI 0.78-1.55), stroke (aRR, 1.47; 95% CI, 1.15-1.88), and Parkinson disease (aRR, 1.88; 95% CI, 1.30-2.58) (Table 4). Dementia and mental health conditions were removed from the final model because of multicollinearity. Use of psychoactive medications before presentation was present in 57% of the cohort (464 patients), and included antidepressants (237 patients [29%]), sleeping medications (142 patients [17%]), anticonvulsants (91 patients [11%]), opioids (89 patients [11%]), antipsychotics (79 patients [10%]), and other medication classes (≤73 patients each [<10% each]). Patients may have been taking multiple medication classes.

**Table 4.** Factors Associated With Risk of Delirium in Older Emergency Department Patients With Coronavirus Disease 2019

Risk factor	Patients, No. (%) (N = 817)	Adjusted RR (95% CI)
Age >75 y	459 (56)	1.51 (1.17-1.95)
Living in nursing home or assisted living	297 (36)	1.23 (0.98-1.55)
Prior psychoactive medications	464 (57)	1.42 (1.11-1.81)
Vision impairment	59 (7)	1.98 (1.54-2.54)
Hearing impairment	54 (7)	1.10 (0.78-1.55)
Stroke or cerebrovascular accident	107 (13)	1.47 (1.15-1.88)
Parkinson disease	23 (3)	1.88 (1.30-2.58)

Abbreviation: RR, relative risk.

Clinical outcomes in this COVID-19 cohort are presented in Table 5. Outcomes significantly associated with delirium after adjustment included hospital admission (aRR, 1.06; 95% CI, 1.02-1.10), any ICU stay (aRR, 1.67; 95% CI, 1.30-2.15), discharge to a rehabilitation facility (aRR, 1.55, 95% CI, 1.07-2.26), and death (aRR, 1.24; 95% CI, 1.00-1.55).

**Table 5.** Outcomes Associated With Delirium in Older Emergency Department Patients With Coronavirus Disease 2019

Outcome	Outcome rate, patients, No./total (%)			RR (95% CI)	
	Overall (N = 817)	Patients with delirium (n = 226)	Patients without delirium (n = 591)	Crude	Adjusted^a^
Admitted to hospital from emergency department	737 (90)	213 (94)	524 (89)	1.06 (1.02-1.11)	1.06 (1.02-1.10)
Any intensive care unit stay^b^	191/737 (26)	72/213 (34)	119/524 (23)	1.49 (1.16-1.90)	1.67 (1.30-2.15)
Received mechanical ventilation^c^	126/191 (66)	42/72 (58)	84/119 (71)	0.83 (0.66 – 1.04)	0.89 (0.71-1.12)
Median hospital length of stay >8 d^b^	353/737 (48)	109/213 (51)	244/524 (47)	1.10 (0.93-1.29)	1.14 (0.97-1.35)
Discharged to					
Home	195 (24)	35 (15)	160 (27)	0.57 (0.41-0.80)	0.77 (0.56-1.07)
Assisted living	21 (3)	8 (4)	13 (2)	1.61 (0.68-3.83)	1.21 (0.49-3.00)
Rehabilitation	111 (14)	41 (18)	70 (12)	1.53 (1.08-2.18)	1.55 (1.07-2.26)
Nursing home	116 (14)	31 (14)	85 (14)	0.95 (0.65-1.40)	0.83 (0.57-1.20)
Hospice	15 (2)	2 (1)	13 (2)	0.40 (0.09-1.77)	0.37 (0.08-1.78)
In-hospital death	238 (29)	84 (37)	154 (26)	1.43 (1.15-1.77)	1.24 (1.00-1.55)

Abbreviation: RR, relative risk.

^a^ Adjusted for age older than 75 years, the presence of 4 or more comorbid conditions, living in nursing home or assisted living, and prior psychoactive medications.

^b^ The sample for any intensive care unit stay and hospital length of stay more than 8 days is 737 patients.

^c^ The sample for receiving mechanical ventilation is 191 patients, the same number of patients requiring intensive care unit care.

## Discussion

In this multicenter retrospective cohort study, 28% of 817 older patients with COVID-19 infection had delirium on arrival to the ED, and delirium was the sixth most common presenting symptom or sign overall. Among patients with delirium, delirium was the main presenting symptom in 16%. Importantly, 37% of patients with delirium did not have typical COVID-19 symptoms of fever or shortness of breath. Factors associated with risk of delirium included older age, prior psychoactive medication use, assisted living or skilled nursing facility residence, vision or hearing impairment, stroke, and Parkinson disease. Delirium at presentation was significantly associated with increased risk for poor hospital outcomes, including ICU stay, discharge to a rehabilitation facility, and death.

The rate of delirium in this cohort is much higher than typically reported in ED studies before COVID-19, which ranged from 7% to 20%,^29^ although the factors associated with risk of delirium are similar to those identified in ED and inpatient research before COVID-19.^7,30,31^ Mounting evidence supports the high occurrence of delirium and other neuropsychiatric manifestations with COVID-19, with previously reported rates of 22% to 33% among hospitalized patients.^19,21^ In another medical record–based study, delirium was reported to be present in 12% of ED patients aged 50 years and older with COVID-19.^19^ Factors potentially contributing to the higher rate of delirium in our study include use of a structured delirium assessment tool at some sites and higher prevalence of comorbid conditions in and older age of our study population. In our cohort, 30% of patients had a prior diagnosis of cognitive impairment or dementia and 13% had a prior history of stroke, compared with 4% and 7%, respectively, in a previous study,^19^ and those groups are at high risk for delirium.^7,31^ The racial and ethnic diversity of our cohort may have contributed to higher rates of delirium because non-White patients hospitalized with COVID-19 may present with higher severity of disease in the US.^32^ It is also possible that the patients may have presented to the ED at a later stage of COVID-19 because of family or clinicians not recognizing delirium as an important symptom, or because of delays in assessment either from fear of exposure to COVID-19 or difficulty accessing medical care.

Commonly cited typical COVID-19 symptoms include fever, cough, shortness of breath, and difficulty breathing.^33^ Fever has been reported to be present in 80% of patients with COVID-19,^33^ but it was present in only 56% of our patients. The absence of typical symptoms or signs is potentially attributable to physiological changes associated with aging. Older adults have a lower basal temperature and an impaired ability to mount a febrile response in the setting of acute infection.^34^ Among patients with delirium in our study, approximately one-third (37%) lacked the typical symptoms and signs of COVID-19 (ie, fever or shortness of breath). Our study demonstrates that clinicians must include COVID-19 in the differential diagnosis of delirium among older adults, regardless of whether they have other symptoms of COVID-19 infection. This is important to avoid missing diagnoses altogether and to better identify severe cases of COVID-19 at high risk for poor outcomes and death. Furthermore, the data from this multicenter study strongly support an immediate revision in CDC guidance on symptom profiles for COVID-19 to include delirium as an important COVID-19–related symptom. Current CDC guidance lists *new confusion* as an emergency warning sign only, not as a presenting symptom in COVID-19. Many centers use the CDC guidance to prioritize screening, testing, and evaluation of presenting patients. By continuing to exclude delirium as a known presenting symptom of COVID-19, many cases will be missed or diagnoses delayed, as is already happening on a wide scale, particularly in older adults.^35,36^

Delirium was associated with adverse outcomes, including ICU stay and death. Thus, delirium can serve as an important marker to identify patients at high risk for poor outcomes. Although our study was unique and innovative in documenting the atypical presentation of COVID-19 in our older adult population, our findings also serve to confirm a recent study^19^ that similarly demonstrated that inpatient delirium among middle-aged and older adults with COVID-19 is associated with ICU stay (adjusted odds ratio, 3.32), mechanical ventilation (adjusted odds ratio, 1.99), and in-hospital death (adjusted odds ratio, 1.75).

### Strengths and Limitations

Strengths of this study include its innovative approach to examining presentations of COVID-19 in a large multicenter cohort, along with identifying major predisposing risk factors and outcomes associated with delirium. The rigorous medical record extraction process across a large cohort involving multiple sites in different states has allowed us to confirm clinical observations from smaller case series. Our study also describes in detail the features and characteristics of prevalent delirium in this cohort of older ED patients with COVID-19.

Several limitations are important to mention. First, we suspect that the prevalent delirium rate of 28% observed in this cohort is an underestimate of the true rate, because we were dependent on medical record documentation where the symptoms are often underreported.^37^ This was a necessary limitation because most EDs do not routinely screen for delirium, and clinical research staff were not allowed in ED settings because of infection control policies during the time of this study. Moreover, many patients with COVID-19 present to the ED in a critical state, necessitating immediate intubation, and ED staff may not have had enough time or resources to assess mental status or obtain collaborative history to confirm a recent change. Less than one-half of our cohort was diagnosed using formal delirium assessment tools. Many cases lacked documentation of baseline mental status, and our approach required a change from baseline to identify delirium. However, we must acknowledge that delirium may have been misclassified by our record review method. Although the medical record reviewers could not be blinded to delirium in their reviews, careful training, standardization, reliability assessments, and adjudication of uncertain ratings were conducted to enhance accuracy and reliability of medical record delirium diagnoses. Second, to create a deidentified data set, the investigators were blinded to site by institutional review board requirements; therefore, we were unable to evaluate any site-specific data. Third, because of the scarcity of testing in some regions, classic imaging findings were accepted for the diagnosis of COVID-19 in this study; however, only 4 of these cases were not confirmed by PCR testing. Fourth, some data, including education, living situation, and socioeconomic status were missing from the record. Fifth, a large proportion of included patients were from assisted living facilities or nursing homes (36%) and/or had previously been diagnosed with dementia (30%). Sixth, enrollment occurred during an 11-week period early in the US pandemic and our study sites were disproportionately distributed in the Northeastern US, which was undergoing a surge in COVID-19 infections at the time. These factors may limit the generalizability of the findings, especially for community-dwelling older adults without cognitive impairment, regions not under surge conditions, and/or during later phases of the pandemic. Seventh, because our study was focused on examining delirium as a presenting symptom, we did not collect data on incident delirium or on preventable or precipitating factors occurring during hospitalization. These will be important areas to evaluate in future research.

## Conclusions

Patients with COVID-19 who present with delirium, either as the main symptom or as one of the presenting symptoms or signs, have worse outcomes, including ICU stay and in-hospital death, than those without delirium. Our study demonstrates that it is critical to recognize that older adults with COVID-19 may present with delirium as the primary or sole symptom. In addition, delirium is an important risk marker to identify patients at high risk for poor outcomes, including death. Future studies will be critical to evaluate the preventable nature of delirium in COVID-19 and the effectiveness of tested intervention strategies, such as the Hospital Elder Life Program^38^ or the ABCDEF bundle,^39^ to reduce the severity and duration of delirium and the occurrence of associated complications. Adding delirium as a common presenting symptom of COVID-19 will keep important cases from being missed and allow earlier identification and management of vulnerable patients at high risk for poor outcomes.
